# Controlling Citrus Huanglongbing: Green Sustainable Development Route Is the Future

**DOI:** 10.3389/fpls.2021.760481

**Published:** 2021-11-15

**Authors:** Xue Li, Huaqin Ruan, Chengqian Zhou, Xiangchun Meng, Wenli Chen

**Affiliations:** ^1^MOE Key Laboratory of Laser Life Science, Institute of Laser Life Science, Guangzhou, China; ^2^Guangdong Provincial Key Laboratory of Laser Life Science, College of Biophotonics, South China Normal University, Guangzhou, China; ^3^Key Laboratory of South Subtropical Fruit Biology and Genetic Resource Utilization (MOA), Guangzhou, China; ^4^Key Laboratory of Tropical and Subtropical Fruit Tree Research, Guangzhou, China; ^5^Institute of Fruit Tree Research, Guangdong Academy of Agricultural Science, Guangzhou, China; ^6^State Key Laboratory of Biocontrol and Guangdong Key Laboratory of Plant Resources, School of Life Sciences, Sun Yat-sen University, Guangzhou, China; ^7^Neuroscience Laboratory, Hugo Moser Research Institute at Kennedy Krieger, Baltimore, MD, United States

**Keywords:** citrus huanglongbing, sustainable development, physical method, antibiotic, antimicrobial peptide, genetically modified technology, nanotechnology, microbial therapy

## Abstract

Huanglongbing (HLB) is the most severe bacterial disease of citrus crops caused by *Candidatus* Liberibacter spp. It causes a reduction in fruit yield, poor fruit quality, and even plants death. Due to the lack of effective medicine, HLB is also called citrus “AIDS.” Currently, it is essential for the prevention and control of HLB to use antibiotics and pesticides while reducing the spread of HLB by cultivating pathogen-free seedlings, removing disease trees, and killing Asian citrus psyllid (ACP). New compounds [e.g., antimicrobial peptides (AMPs) and nanoemulsions] with higher effectiveness and less toxicity were also found and they have made significant achievements. However, further evaluation is required before these new antimicrobial agents can be used commercially. In this review, we mainly introduced the current strategies from the aspects of physical, chemical, and biological and discussed their environmental impacts. We also proposed a green and ecological strategy for controlling HLB basing on the existing methods and previous research results.

## Introduction

Huanglongbing (HLB), named for the leaf yellowing of diseased citrus, was first discovered in the Chaoshan area of Guangdong Province, China in the 1910s (Reinking, 1919). The typical symptoms of HLB-infected citrus are leaf yellowing, and roots rot in varying degrees (Bové and Barros, 2006). Even worse, HLB-affected fruits become smaller, taste sour, and bitter, and are prone to abscission (Bassanezi et al., 2009). HLB has caused a huge financial loss in the citrus industry. With the deepening of globalization, HLB has spread from Asia to Africa and the Americas (Faghihi et al., 2010; Lopes et al., 2010; Bassanezi et al., 2020). It has affected major citrus-producing areas and severely hindered the development of the citrus industry. Due to HLB, 7.4 million trees were lost in Guangxi, China alone in 2020, and more than 10 million diseased trees were destroyed all over China each year (Zhou, 2020). Many countries, namely China, the United States, and Brazil, have attached great importance to the prevention and control of HLB and invested heavily in related fields. How to defeat HLB has long been a common problem faced by all countries.

It goes through the process of the nematode from fusarium to a virus to mycoplasma in the understanding of HLB pathogens. With the maturity of microscope technology, the peptidoglycan layer was observed under the electron microscope between the outer membrane and inner membrane of the HLB pathogen, which proved that it belongs to Gram-negative bacteria (Garnier et al., 1984). At present, it is commonly believed that the HLB pathogen belongs to *Candidatus* Liberibacter spp. of the *α-proteobacteria*, mainly divided into *Candidatus* Liberibacter asiaticus (*Ca*.Las), *Candidatus* Liberibacter africanus (*Ca*.Laf), and *Candidatus* Liberibacter americanus (*Ca*.Lam) according to regionality, heat sensitivity, and 16S rDNA (Bové, 1974; Bové and Barros, 2006). Among them, *Ca*.Las is the most pathogenic and widely distributed species. HLB caused by *Ca*.Las has been reported in more than 20 countries and regions (Sechler et al., 2009; Hartung et al., 2010).

*Candidatus* Liberibacter asiaticus can infect almost all parts of the plant, but its distribution varies in different tissues (Johnson et al., 2014; Hajeri and Yokomi, 2019). The bacterial titer of leaves and stems is higher than in other parts of the plant. The Asian citrus psyllid (ACP) feeds on the phloem sap of citrus trees. *Ca*.Las enters into the body of ACP and multiplies in the insect gut by this way. Then, they spread from the gut to the salivary gland and gonad by blood circulation. Eventually, *Ca*.Las is transmitted to new hosts during ACP sucks sap from healthy plants (Grafton-Cardwell et al., 2013; Kruse et al., 2019). Bacteria deliver effector proteins into host cells through many kinds of secretion systems. *Ca*.Las only encodes genes for type I secretion system (T1SS) and Sec-dependent secretion system, whereas the genes of other secretion systems are lacking (Duan et al., 2009; Wang et al., 2017). The gene encoding a protein of the serralysin family was found next to the T1SS of *Ca*.Las. The previous study has shown that this protein played an important role in the bacteria against host defense. It also has strongly expressed in citrus phloem (Felfoldi et al., 2009; Faghihi et al., 2010; Cong et al., 2012). Thus, this protein may be a potential effector protein. Virulence factors of *Ca*.Las are secreted to the phloem elements or companion cells of citrus primarily by the Sec-dependent secretion system (Samiksha et al., 2016; Wang et al., 2017). The Sec-dependent secretory protein 1 (SDE1) has been identified in detail. It can suppress host defense response to promote bacterial invasion and colonization by interacting with citrus papain proteases (Marco et al., 2016). In addition, Zhang et al. (2020) found that the secretory protein CLIBASIA_04405 could inhibit the hypersensitivity and H_2_O_2_ accumulation in tobacco. The discovery of secretory proteins provides the basis for studying the pathogenicity mechanism of *Ca*.Las. Because most of *Ca*.Las cannot be purified and cultured *in vitro*, studies that the microbial-host molecular level has been greatly limited.

The struggle against HLB has been persisted for more than one century. Numerous scholars have made great contributions to the research of HLB during this period (Bassanezi et al., 2020). However, there is no cure. Some medicines have been widely used to ensure the healthy development of the citrus industry, such as antibiotics, pesticides, and immune inducers, which have achieved remarkable results (Zhang et al., 2011; Stockwell and Duffy, 2012). In 2018, the United States Department of Agriculture and Environmental Protection approved the combination of terramycin and streptomycin to control citrus HLB (Hijaz et al., 2021). Besides, cutting off the transmission route is also an essential step for controlling HLB (Zheng et al., 2018). Regrettably, these methods cannot completely get rid of the pathogen. With the increasing awareness of human environmental protection, the trend of biological control of HLB is becoming increasingly popular. The identification of new antimicrobial peptides (AMPs) and the application of transgenic technology have provided new hope for the control of HLB.

**FIGURE 1:**
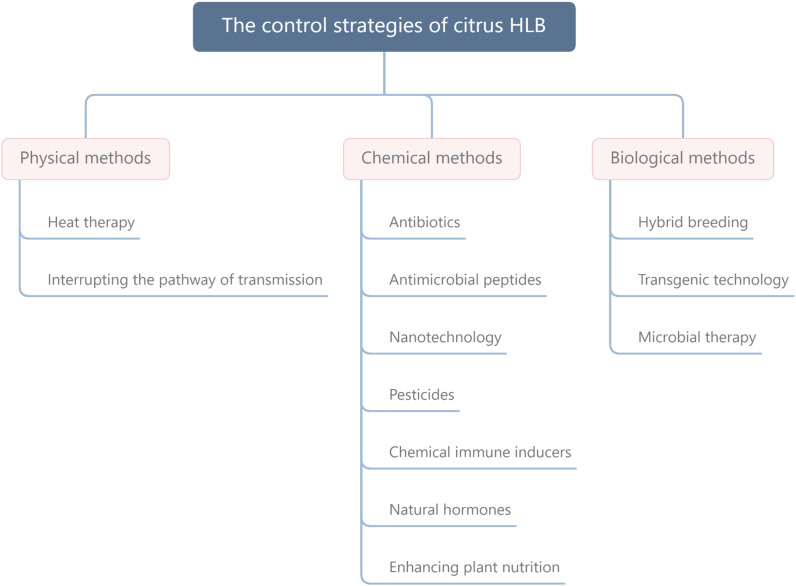
The control strategies of citrus HLB mainly include physical methods, chemical methods, and biological methods. HLB, huanglongbing.

**FIGURE 2:**
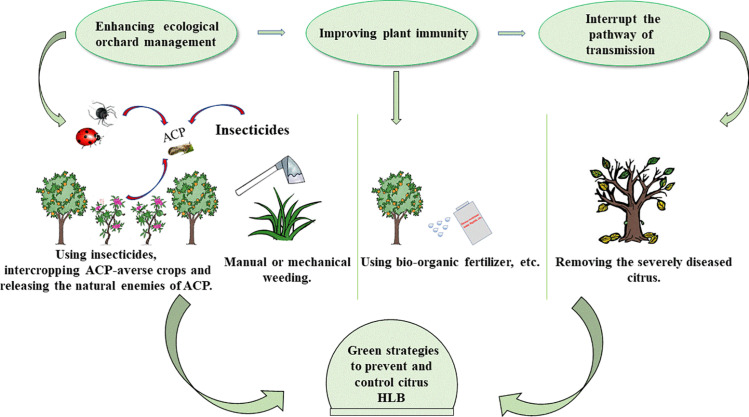
Green strategies to prevent and control HLB. There are mainly three steps: first of all, enhancing the management of orchards by manual or mechanical weeding, intercropping ACP-averse crops, and releasing the natural enemies of ACP. Secondly, improving plant immunity. Using bio-organic fertilizer, spraying metabolic photosynthetic accelerators, and stress-resistant ionic liquids to enhance plant immunity. Finally, removing the severely diseased citrus to cutoff HLB from the source. HLB, Huanglongbing; ACP, Asian citrus psyllid.

Fosthiazate (FOS) is a commercial organophosphorus pesticide that can effectively kill nematodes, and cupric-ammonium complex (CAC) is a broad-spectrum fungicide with low toxicity. Our laboratory found that the combined therapy of CAC and FOS was effective for HLB (Duan et al., 2021). Accumulated pieces of evidence show that a single measure is not strong enough to control HLB, and management of HLB requires integrating prevention with control. In this article, we summarize and discuss the HLB control methods, such as physical methods, chemical methods, and biological methods (Figure 1), hope to provide a reference for the comprehensive administration of HLB. In addition, we also propose an environmentally friendly strategy for controlling HLB (Figure 2).

## Physical Methods

### Heat Therapy

Heat therapy has long been used in the field of plant disease management because high temperatures can cause cell lysis and thus kill bacteria (Schuster et al., 1973). In apple trees, the rubberwood virus can be eliminated by heat therapy (Campbell, 1961). In the 1960s, heat therapy has been utilized to treat citrus HLB by Kongxiang Lin (Lin and Luo, 1965). Nowadays, this approach has already been shown productive and widely used in the study of HLB. Among the three pathogenic bacteria, *Ca*.Las is able to tolerate temperatures above 35°C (Bové, 1974). Therefore, a temperature above 40°C is usually selected as a heat treatment condition in the greenhouse. We compared the results from different investigators and found that the symptoms of HLB-infected trees were alleviated to a certain extent after continuous treatment between 40 and 50°C. The *Ca*.Las titer decreased to undetectable levels and most of the trees restored health in treated groups compared with untreated groups (Hoffman et al., 2013; Fan et al., 2016; Munir et al., 2018). All these experimental results show that heat therapy is effective against HLB. Although it has been proposed that heat stress may damage the citrus plants, many studies show that it can enhance plant metabolism and thus increase plant growth vigor and productivity (Lin and Luo, 1965). However, the presence of germs in citrus roots results in difficulty treating in wild. Limited by human and material resources, heat therapy is currently difficult to be applied in orchards.

### Interrupting the Pathway of Transmission

The two major transmission pathways of HLB are grafting diseased branches and spreading by ACP. Therefore, interrupting the pathway of transmission can effectively reduce the spread of HLB. The “three-step method” has been proved to be effective in reducing the spread of HLB (Lin, 1963), which is still adopted by countries all over the world.

#### The Cultivation of Pathogen-Free Seedlings and Uprooting Diseased Trees

Using the seedlings which are acquired from diseased trees or polluted breeding bases is responsible for the crazy spread of HLB in citrus. So, building a citrus pathogen-free breeding system is important for containing the transmission of HLB (Navarro, 1992). Presently, there are mainly three kinds of acquiring pathogen-free seedlings: (1) heat treatment: the pathogen can be fully cleared in quality buds by treating for consecutive 8 h and total for at least 40 h at 40−50°C (Hoffman et al., 2013; Fan et al., 2016). (2) Stem tips culture: plant apex tissues containing little or no germs and viruses are the ideal material to obtain pathogen-free seedlings (Prasad et al., 2012; Juárez et al., 2015). This technology has become one of the main techniques to culture pathogen-free seedlings. (3) Micrografting: micrografting technology is a method that combines stem tips culture and grafting, and culturing pathogen-free seedlings are mainly achieved by grafting pathogen-free stem tips onto a sterile plate (Starrantino and Caruso, 1988; Abbas et al., 2008). It is worth noting that every detoxification technology is inseparable from heat treatment. The pathogen-free seedlings must be identified (phenotypic identification or qPCR detection) before the promotion and application (Arredondo Valdés et al., 2016). Because all parts of diseased citrus may carry the pathogen, it is ineffective to control HLB only by cutting diseased branches (Lopes et al., 2007). Therefore, suspected diseased trees should be detected as soon as once they were found in the orchard. Once confirmed, it needs to uproot the HLB-infected trees, and the stumps should be perfused with herbicide and covered with film to prevent regeneration. In theory, the primary location should not be re-implanted for a number of years. In addition, strengthening orchard management can effectively reduce the burst rate of HLB (Bové and Barros, 2006). The orchards with poor management are more likely to relapse HLB than a well-managed orchard.

#### Controlling Asian Citrus Psyllid

Asian citrus psyllid is another major transmission medium of HLB, so controlling ACP is essential for preventing HLB (Grafton-Cardwell et al., 2013; Bassanezi et al., 2020; Li et al., 2020). ACP is easily attracted to yellow objects and methyl salicylate. Yielding lots of methyl salicylate and leaf yellowing further attract ACP to suck the sap of diseased trees results in the spread of HLB (Tiwari et al., 2010). Thus, it is necessary to eliminate ACP in time to prevent the spread of HLB after finding diseased trees (Grafton-Cardwell et al., 2013). Currently, the most frequent method of reducing ACP is through the use of chemical drugs, such as pyriproxyfen (Boina et al., 2010), imidacloprid (Katsuya et al., 2010), horticultural mineral oil (Teck et al., 2012), and aldicarb (Qureshi and Stansly, 2010). It was found that the cytochrome P450 monooxygenases (*CYP4*) genes were associated with insecticides resistance in ACP. The *CYP4* expression of *Ca*.Las-infected ACP was lower than non-infected ACP, this showed that infected ACP was more sensitive to insecticides (Tiwari et al., 2011a,b). However, chemical drugs are unfriendly to the environment, other beneficial insects, and human health. Due to the short life cycle and strong reproductive capacity of ACP, the drugs are regularly applied every year. The resistance of ACP may be enhanced under intensive application of insecticides and thus lead to the weakening effect of the drugs (Liu and Tsai, 2000; Wenninger and Hall, 2007). Hence, compared with chemical drugs, biological control of ACP has greater development prospects, mainly including (1) entomopathogenic fungal therapy: *Isaria fumosorosea* (Meyer et al., 2008; Hoy et al., 2010; Hunter et al., 2011), *Hirsutella citriformis* (Meyer et al., 2007; Casique-Valdes et al., 2011), and other fungi are known to be able to infect ACP and also powerful candidates for biocontrol. (2) Parasite therapy: *Tamarixia radiata* and *Psyllaephagus* are two important ACP parasitic insects (Waterston, 1922; Hoy, 2001) that can be used to control ACP populations. (3) Insect viruses therapy: insect viruses are also candidates for biocontrol of ACP. For example, *Diaphorina citri-associated C virus*, *Diaphorina citri picorna-like virus*, and *Diaphorina citri flavi-like virus* are expected to be potential vectors for delivering RNA interference (RNAi) directly to ACP instead of *Citrus tristeza virus* (CTV) vector system (Britt et al., 2020). (4) Natural enemy predation therapy: ladybugs, spiders, and syrphid flies are natural enemies of ACP (Qureshi and Stansly, 2009; Shivankar and Rao, 2010). To some extent, releasing these natural enemies is also in favor of limiting ACP population growth. Subsequently, it was found that guava leaves and their volatiles (Onagbola et al., 2011), lavender essential oil, rose essential oil, tea tree essential oil, rutin essential oil, and other natural organic compounds exerted a repellent effect on ACP (Tiwari et al., 2010). Therefore, intercropping citrus with ACP-averse crops is also a method to reduce HLB transmission. Of course, biological control of ACP is not a highly effective control method. It is not highly reliable, and it can be influenced by so many other factors. Organic growers might use it but conventional growers cannot rely solely on biological control. Using insecticides is the cost-effective and most efficient strategy for controlling ACP (Li et al., 2020). To reduce the frequency of use of insecticides and mitigate the resistance of ACP, we propose adopting comprehensive management strategies that include releasing ACP natural enemies, intercropping ACP-averse crops, and using insecticides after attracting and aggregating ACP by the yellow sticky board or transgenic citrus.

Depending on interrupting the pathway of transmission can only stop the spread of HLB instead of curing it.

## Chemical Methods

### Antibiotics

Since antibiotics were discovered, they have made a significant contribution to the control of bacterial diseases in plants (Stockwell and Duffy, 2012). Tetracycline has been widely used to treat HLB in various countries as early as the 1970s (Su and Chang, 1974). However, it was later replaced by other antibiotics, such as penicillin, streptomycin, and terramycin, according to the poor results of assessment for tetracycline (Zhang et al., 2012, 2014; Hu et al., 2018). In fact, co-treatment with two antibiotics worked better than that of either antibiotic alone. Zhang et al. (2011) found that the regeneration rate of plants after stems of HLB-infected trees was immersed in penicillin (100 μg/ml)-streptomycin (10 μg/ml) for 4 h was higher than other treatments. Today, hygromycin and streptomycin have been allowed to be used commercially (Hijaz et al., 2021). To investigate the transport process of antibiotics in citrus, fluorescence-labeled penicillin was injected into citrus and observed with fluorescence microscopy (Carl Zeiss Microscopy GmbH, Göttingen, Germany). The fluorescence was detected in tissues of the plant and ACP (Killiny et al., 2019). The hygromycin also was observed in xylem- and phloem-related tissues after 24 h when trunk injection. It was detected in leaves after 3 days, and only very little was detected in roots (Hijaz et al., 2020, 2021). These results suggest that antibiotics are transported to various organs following the “vascular system” of the plant, and the transport efficiency varies depending on the tissues. The influence of antibiotics on the plants’ growth and fruit quality is the major concern problem. It was shown that the application of penicillin and hygromycin increased N, P, K, S, and Zn contents in leaves, soluble organic matter content in fruits, and the nutritional status of citrus trees (Zhang M. et al., 2021). Although antibiotics brought convenience to agricultural production, their usage was controversial. The first point was the costs and the damage to plants of antibiotics (Zhang et al., 2014; Li et al., 2020). On the other hand, antibiotics may also increase bacterial resistance and cause superbugs (Bryson and Demerec, 1955). In addition, trunk injection leads to a better effect of antibiotics than traditional spraying. But it is very difficult in actual practice due to the large costs of human and material resources. The development of an efficient and convenient trunk injection method will play a key role in the treatment of HLB.

### Antibacterial Peptides

Antimicrobial peptides are small-molecule proteins with extensive antimicrobial activity secreted by the host and have a regulatory effect on immune response (Koczulla and Bals, 2003). *Ca*.Las mainly infects *Citrus* and its related genera of *Rutaceae*, but different varieties have different sensitivity to HLB (Folimonova et al., 2009). When infected, severe symptoms of leaves yellowing and easily fall off occurred in most commercial citrus whereas Citrus medica (*C. medica*) and *Poncirus trifoliata* (*P. trifoliata*) with tolerance to HLB had mild or no symptoms and normal growth and development (Albrecht and Bowman, 2011, 2012). Huang et al. (2021) have identified a short antimicrobial peptide (SAMP) that was only present in HLB-tolerant citrus by analysis of small RNA and mRNA between HLB-susceptible and HLB-tolerant citrus cultivars. Compared with antibiotics, the greatest advantages of SAMP were its thermal stability and high efficiency, which made it more suitable for practical application. Moreover, the SAMP was extremely easy to be degraded by pepsin and had higher safety (Wang et al., 2021). AMPs have been studied for a long time in citrus HLB control. Stover et al. (2013) used the *Agrobacterium tumefaciens*, *Sinorhizobium meliloti*, and *Xanthomonas citri* subsp. *citri* as experimental materials to explore the bactericidal effect of 44 broad-spectrum AMPs. It was also bactericidal property at lower concentrations (1 μM). AMPs as safe and efficient antibacterial agents are suitable alternatives to antibiotics in agricultural production. However, the studies of AMPs are primarily focused on the level of basic research, and also the preparation cost of AMPs is too high for its commercial applications in controlling HLB.

### Nanotechnology

Nanotechnology is an interdisciplinary technology with a wide range of applications. In the agricultural field, it is mainly used for disease management and control (Rai and Ingle, 2012) and includes (1) diagnosis of the crop diseases by using nanosensors (Gitaitis and Walcott, 2007). (2) Metal nanomaterials control crop diseases. (3) To improve drug utilization through nanoemulsion complexes. Spraying on the leaves is the major form of drugs administered in the field. However, the drug uptake efficiency of plants is greatly hindered due to the presence of the hydrophobic wax layer on the surface of citrus leaves. Due to the small size, nanoparticles can modify the property of drugs and enhance the absorption rate of plants for drugs. In recent years, nanotechnology has also been applied to treat citrus diseases and achieved good results. Young et al. (2018) applied ZnO–nCuSi with the low phytotoxic composite to treat citrus. Field experiments showed that ZnO–nCuSi had strong antibacterial activity to effectively control citrus canker. Kumar et al. (2018) found that nano-ZnO-2S albumin protein was able to significantly inhibit the growth of *Ca*.Las. Although some metal agents (such as ZnO) have been considered as fertilizers for agricultural production, they do not play the biggest function due to their low solubility. This problem was not solved until the advent of nanoscale metal agents, which have high solubility and larger specific surface area, can catalyze the production of singlet oxygen and show strong bactericidal ability. Currently, nanoscale ZnO and silver nanoparticles (AgNPs) have been proved that not only have good bactericidal effects but effectively enhance plant resistance to fungal diseases, such as citrus scab and melanosis too (Graham et al., 2016; Stephano-Hornedo et al., 2020). Compared with the treatment of β-lactam antibiotics, the content of starch granules present in phloem sieve tubes and the *Ca*.Las titers were also significantly reduced after being treated by using AgNPs in HLB-infected trees (Stephano-Hornedo et al., 2020). Nanotechnology has brought new ideas for the prevention and treatment of HLB. Nanocomposites not only improve the utilization of drugs, they reduce the usage of drugs and also alleviate environmental pressure. Because we successfully silenced the *NPR1* gene using AuNPs carrying siRNA*_NPR1_* (Lei et al., 2020). Our laboratory is developing a nano-genetic technology to activate a defense system for improving resistance by transferring resistant genes to hosts through nanomaterials, hoping to contribute to the prevention and treatment of HLB.

### Pesticides

Citrus trees often suffer damage from nematodes (Cobb, 1914; Verdejo-Lucas and Mckenry, 2004). So far, more than 40 species of citrus parasitic nematodes have been identified worldwide, with two main common species: *Meloidogyne* and *Pratylenchus* spp. (Govaerts et al., 2007; Collange et al., 2011). The nematodes not only damage citrus roots and reduce new roots growth, but they can also affect roots development and the ability to absorb nutrients result in the decreased ability to resist diseases (Damadzadeh and Maafi, 2008). It was well known that sieve tube occlusion of HLB-infected citrus caused difficulty in transporting organic matter from leaves to the roots. The nematode colonization was a double blow to roots and thus making symptoms worse (Pourreza et al., 2016). Our study also found the presence of *Tylenchulus semipenetrans* juveniles in the roots of heavily diseased citrus (Duan et al., 2021). Therefore, controlling nematodes is also a necessary step in the treatment of HLB. Some herbicides are also commonly used in agricultural management, such as glyphosate (Duke and Powles, 2010), paraquat (Manning-Bog et al., 2002), atrazine (Hayes, 2002), and FOS (Duan et al., 2021). The use of pesticides provides conveniences in orchard management while safety concerns also cannot be ignored. For instance, the accumulation of pesticides can destroy the ecological balance and endanger human body health. Therefore, it is better to adopt hand or mechanical weeding and the way of soil fumigation and deep tillage to reduce nematodes during the process of HLB prevention and control (Eden and Stirlingl, 2008). In addition, the use of pesticides should be reduced, and organic fertilizers should be properly applied to restore the soil condition.

### Chemical Immune Inducers

Systemic acquired resistance (SAR) is a crucial defense mechanism against pathogen invasion mediated by salicylic acid (SA) in plants (Mou et al., 2003). SA is one of the important signaling molecules in the SAR pathway. A large amount of methyl salicylate, which can be transformed into SA under the action of SA methyltransferase, is rapidly synthesized when *Ca*.Las invades citrus. The expression of pathogen-associated proteins (*PRs*) induced by SA can improve the disease resistance of plants (Kumar and Klessig, 2008; Herrera-Vásquez et al., 2015). However, the genome annotation results showed that there were genes encoding SA hydroxylase in *Ca*.Las, *Ca*.Laf, and *Ca*.Lam (Duan et al., 2009; Lin et al., 2013; Katoh et al., 2014). It can degrade SA to hydroxyl SA which cannot induce the expression of *PRs*, and thus reduce the disease resistance of the plants (Li et al., 2017). Some exogenous chemical reagents have the function of activating plant immunity and can be used as a substitute for SA, such as imidacloprid, β-aminobutyric acid, 2,3-benzothiadiazole, and 2,6-dichloroisoniazid. These immune activators are more stable than SA and not easily degraded by SA hydroxylase secreted by bacteria. It has been shown that surface spraying or trunk injection immune inducers could induce *PRs* expression and enhance plant resistance for a period of time (Francis et al., 2009; Graham and Myers, 2013; Li et al., 2016; Palmer et al., 2019). Field experiments showed that these immune inducers could also control the development of HLB and had a positive impact on citrus yield and fruit quality (Li et al., 2016).

### Natural Hormones

Natural hormones are also important regulators for plant growth and development (Yokota, 1997). High brassinosteroid (HBR), a kind of brassinosteroids (BRs), is a natural hormone that is widely distributed in pollen, seeds, stems, and leaves of plants and plays an important regulatory role in plant growth metabolism (Grove et al., 1979; Bishop and Takao, 2001). BRs can not only help plants cope with various extreme environmental stresses (Divi et al., 2010; Xia et al., 2011) but also improve plant resistance to pathogens (Ali et al., 2013). HBR was used to treat 2-year-old diseased citrus in the greenhouse in Cuba, the results showed that the symptoms were significantly improved and the bacterial titer of diseased citrus even decreased to an undetectable level (Canales et al., 2016). Melatonin is also an important plant regulator of inhibition effect on *Ca.*Las (Nehela and Killiny, 2020). In addition, other natural hormones also play a key role in host response under biological or non-biological stress, such as SA, jasmonic acid (JA), and ethylene (ET) (Mur et al., 2006; Bari and Jones, 2009). Other plant metabolites may also have directly or indirectly influenced the immune regulation against HLB. However, the regulatory mechanisms of most metabolites are unknown and need further research. Although these immune activators improve the nutritional status of citrus, the effect of immune inducers is negatively correlated with the age and disease severity of citrus trees (Li et al., 2016). Therefore, immune activators can be used as adjuvants to prevent HLB.

### Enhancing Plant Nutrition

The early symptoms caused by HLB are similar to those caused by the lack of trace elements (Mattos et al., 2020). Zn and P deficiencies could also promote HLB occurrence (Zhao et al., 2013). Sieve tube occlusion caused by *Ca*.Las can limit the transport and absorption of nutrients. Therefore, an additional nutrient supply can alleviate HLB symptoms and prolong plant life (Pustika et al., 2008). Studies have indicated that the N, Mn, Zn, and SA contents were significantly higher in the leaf of nutrient supply trees than the no-nutrient supply trees (Shen et al., 2013). The microbiota has a regulatory effect on plant growth and development and that the microbial abundance could be changed by using Zn-containing additive (Zhang et al., 2016). This further demonstrated the important function of increasing additional nutrients in plant growth. Because the effect is related to the level of disease, additional nutrient supply is more effective when it is applied at the earlier phase of infection. Certainly, only enhancing nutrition does not effectively control HLB, or even bacterial titer can be increased in the short term (Gottwald et al., 2012). Therefore, it should be treated with the simultaneous use of nutrients (e.g., nitrogen fertilizer and mixed fertilizer) and defense activators for the early control of HLB (Li et al., 2019; Zhang et al., 2019).

Since there are no specific drugs that can cure HLB, the overuse of chemical drugs may cause a series of safety problems in the environment and biology. So biological method is the general trend of the prevention and treatment of HLB and also has made a great contribution to the control of other agricultural diseases.

## Biological Methods

### Hybrid Breeding

New varieties with excellent traits were obtained by hybrid breeding (Ohgawara et al., 1994; Viloria and Grosser, 2005; Grosser et al., 2007, Grosser and Gmitter, 2011, and this technology has been often used to improve the stress resistance of citrus (Grosser, 2003). The resistance traits obtained through the somatic hybridization technique can be stably inherited to offspring. However, the leaf malformation was found in a new plant that fused the somatic cells of *Citrus sinensis* and *Murraya exotica* (Grosser and Olivares-Fuster, 2000). Reproductive isolation between citrus and its distant relatives results in these materials being unusable. Disease-resistant materials were obtained primarily through rootstock breeding in citrus, because of the long breeding cycle and complex operations of traditional breeding (Bowman and Joubert, 2020). *P. trifoliata*, *C. medica*, *Citrus limon* (*C. limon*), and other *citrus* germplasms that have HLB tolerance are the main rootstock source for citrus (Miles et al., 2017). Because rootstock affects the life, nutrition, and growth of citrus (Sabatino et al., 2019), different regions should choose suitable rootstock varieties. The plant traits of *P. trifoliata* and *C. sunki* are compatible so that their hybrids can obtain the characteristics of HLB tolerance and have mild disease symptoms when infected (Ramadugu et al., 2016; Curtolo et al., 2020). The application of hybrids with HLB tolerance is conducive to alleviating the pressure caused by HLB in the short term. These results not only indicate that there are defense mechanisms against *Ca*.Las in HLB-tolerant plants but also tolerance characteristics can be inherited. Curtolo et al. (2020) revealed that the HLB tolerance mechanisms were related to the downregulation of gibberellin (GA) and the enhancement of the cell wall. The sensitivity of different plants to HLB may be related to the difference in phloem cell activity (Fan et al., 2012). These results are crucial for identifying key tolerance-associated genes, and candidate genes are expected to construct HLB-tolerant citrus through genetic engineering technology.

### Transgenic Technology

Transgenic technology was widely used to improve varieties in agriculture and animal husbandry (Mitchell and Sheehy, 2000). The progress of cultivating new plants is slow through traditional hybrid breeding technology because of the long breeding cycle of citrus. Transgenic technology can not only shorten the production cycle but also break the incompatibility barrier of distant hybridization, and also keep the genotypic and phenotypic integrity of transgenic citrus. Recently, Endo et al. (2020) developed a fast-track breeding system to transfer the CTV resistance of trifoliate orange into citrus germplasm, which greatly shortened the breeding cycle and was in favor of the application of transgenic technology.

The non-expresser of pathogenesis related protein 1 (*NPR1*), as a SA receptor, plays an important role in the SAR pathway (Mou et al., 2003; Spoel, 2003). Overexpression of *AtNPR1* (the *NPR1* of *Arabidopsis thaliana*) enhanced the disease resistance in transgenic crops, such as rice, apple (Chern et al., 2005; Malnoy et al., 2007; Sajad et al., 2017), and sweet orange (Zhang et al., 2010; Boscariol-Camargo et al., 2016). It was later found that transgenic citrus plants overexpressing *AtNPR1* exhibited tolerance to HLB (Dutt et al., 2015). Transgenic plants had lower *Ca.*Las titer and better growth state than non-transgenic plants under HLB stress (Dutt et al., 2015; Caserta et al., 2020). Citrus thionein is a known antibacterial agent, and transgenic citrus overexpressing thionein performed better under HLB stress compared to the non-transgenic citrus (Hao et al., 2016). The AMP genes previously mentioned are strong candidates for the construction of transgenic plants. For instance, transgenic plants overexpressing *D4E1* obtained HLB tolerance because synthetic peptide *D4E1* could lead to bacterial cell membrane lysis (Attílio et al., 2013; Caserta et al., 2020). However, transgenic plants can only increase tolerance to HLB, but cannot solve the problem fundamentally.

Transgenic technology is also applied to control ACP. *Bacillus thuringiensis* (*Bt*) toxin can effectively kill lepidopteran insects (Naranjo, 2011). Transgenic *Bt* citrus greatly reduced the use of pesticides and suppressed pests’ growth. Some volatile organic compounds, such as the volatile β-caryophyllene, could repel ACP by affecting the behavior of pests, and the transgenic citrus had a low attraction for ACP to reduce the spread of HLB (Alquézar et al., 2017). Thus, it is considered an environmentally friendly way of controlling pests, and there is no obvious phenotypic change in transgenic citrus. However, transgenic products are facing great controversy. Since exogenous genes are expressed in fruits, the impact on human health is unknown. Especially in the EU and Japan, people generally oppose the application of this technology. In the future, solving and balancing this problem is inevitable (Grosser et al., 2009).

*Citrus tristeza virus* is a ubiquitous virus in citrus that can enter the ACP body by consuming sap. RNAi technology based on CTV has also become a new method for controlling pests. This transient expression vector is very stable and therefore considered for citrus HLB control (Folimonov et al., 2007). At present, some progress has been made in the field. Key genes involved in metabolism and growth are silenced in ACP when CTV-RNAi is ingested. Reducing the expression of the abnormal wing disc (*Awd*) gene could not only cause ACP malformation but also increase mortality of adults (Hajeri et al., 2014). Besides, the ACP can be killed by insecticides, whereas this may lead to resistance to drugs. Thus, when *CYP4* was silenced, the resistance would be reduced and allow insecticides to act at high potency (Killiny et al., 2014). CTV-t phytoene desaturase-silenced (PDS) plants can attract and gather ACP when planted in orchards. It is convenient to use insecticides to kill ACP. Just CTV-tPDS trees may appear photo-bleaching phenotype (Killiny et al., 2021). The application of a CTV-based vector system is much mature and simpler to apply than insect-virus-based vector technology. Citrus nurseries can start the seedling already inoculated with a CTV-based vector hence the whole new orchard is pre-treated with CTV-based biological control. In addition, a CTV-based vector can be applied to the existing trees in the field without the need to replant the whole orchard. It can be done by graft inoculating the existing trees with plant material carrying a CTV-based vector. On the other hand, insect-virus-based vectors are still under research, and researchers have no idea how to introduce the insect virus safely in the environment. How these ACP-insect viruses going to impact the other beneficial insects is untested territory.

### Microbial Therapy

Soil is a natural large microbial storage, and plant roots are the major sites of microbial interaction (Zhang K. et al., 2021). Improving soil biodiversity is beneficial to strengthen the interaction between soil function and biological elements (Artursson et al., 2006). The benign cycle of root microbial flora is conducive to both improving the growth environment of citrus roots and the prevention and control of HLB (Trivedi et al., 2010). Compared to HLB-infected trees, there are lots of beneficial bacteria, such as *Burkholderia* spp. and *S. meliloti*, in the rhizosphere soil of healthy citrus. These beneficial bacteria can both compete with harmful bacteria and regulate the metabolic activities of citrus roots by inducing the secretion of secondary metabolites (Zhang et al., 2017). There are some seemingly healthy trees in diseased citrus orchards, which are called HLB “escape trees.” Beneficial bacteria with antibacterial ability were also isolated from these citrus roots (Riera et al., 2017). These results further demonstrate that beneficial bacteria are essential for plant growth and health. A study showed that *Burkholderia* spp. that colonize host roots could increase the expression levels of SA pathway-related genes to improve the antiviral ability of plants (Zhang et al., 2017). According to the characteristics, microbial agents that contain actinobacteria, yeast, rhizobium, other beneficial bacteria, and plant growth regulators have been developed and applied to control HLB. The harm of microbial agents is much smaller than chemical fertilizers, which can avoid soil hardness and fusarium root rot caused by excessive use of drugs and fertilizers.

## Conclusion and Perspective

Nowadays, to protect the environment, countries all over the world are paying more and more attention to sustainable agricultural development. Therefore, a combination of the above, we propose a more environmentally sustainable route for the control of HLB (Figure 2). First of all, it is imperative to enhance orchard management by green and integrated approach that includes hand or mechanical weeding, suppressing ACP in combination with chemical and biological methods, such as the use of insecticides and the release of pests natural enemies. In addition, corresponding treatment measures should be taken when other major citrus diseases are present. Secondly, strategies, such as using bio-organic fertilizer and spraying immune activators that improve the plants’ growth environment and enhance plant immunity, have a great promise for controlling HLB. Finally, removal of severely diseased citrus is key to reducing transmission because the effect of medications is guided by the disease severity of HLB-infected citrus. We hope these strategies are useful for HLB.

Since the 21st century, rapid growth in science technologies has also promoted the development of HLB-related research. Transgenic technology and nanotechnology as green strategies have become a hotspot attracting many researchers worldwide. However, lacking HLB resistance genes is the biggest challenge for the application of transgenic technology. But global citrus resources are very abundant and most of them have not been systematically evaluated. It cannot be concluded that resistance genes are absent altogether. In fact, controlling HLB is still a challenging task for investigators and planters who have to face.

**FIGURE 3:**
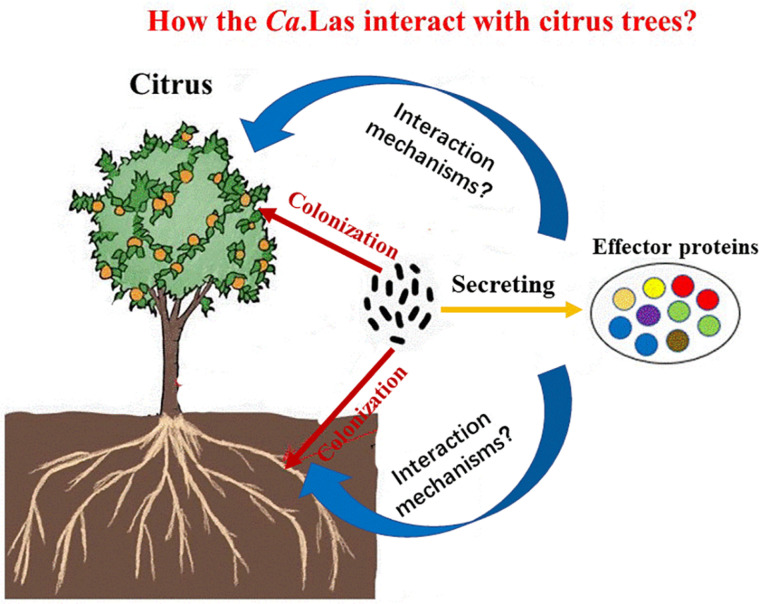
The interaction process between citrus and *Candidatus* Liberibacter asiaticus (*Ca.*Las). *Ca.*Las can more easily colonize in roots, stems, and leaves of citrus through effector proteins. To analyze the interaction mechanism of plant and pathogen is conducive to promote the development of HLB prevention and control. HLB, Huanglongbing.

In our opinion, future research should focus on two key points, such as (1) the interaction mechanisms of plant–pathogen (Figure 3). Studying the physiological and biochemical changes of diseased trees is important to clarify the pathogenesis of HLB and search for efficient treatment strategies. It is also favorable for the targeted transport of nanoparticles in the host and facilitates the applications of nanotechnology. (2) Screening HLB-tolerant plants and resistance genes. This is a critical step in resistance breeding. Driven by the government policies, the development of HLB has largely been inhibited by current prevention and control strategies, and we believe that this problem will be overcome in the future.

## Author Contributions

XL and HR: conceptualization and compilation of data and designing of figures. XL, HR, and WC: writing part and proofreading. XL, XM, and CZ: language polishing. All authors contributed to the article and approved the submitted version.

## Conflict of Interest

The authors declare that the research was conducted in the absence of any commercial or financial relationships that could be construed as a potential conflict of interest.

## Publisher’s Note

All claims expressed in this article are solely those of the authors and do not necessarily represent those of their affiliated organizations, or those of the publisher, the editors and the reviewers. Any product that may be evaluated in this article, or claim that may be made by its manufacturer, is not guaranteed or endorsed by the publisher.
